# Biofilm formation on polyetheretherketone and titanium surfaces

**DOI:** 10.1002/cre2.205

**Published:** 2019-06-13

**Authors:** Sargon Barkarmo, Daniel Longhorn, Kiran Leer, Carina B. Johansson, Victoria Stenport, Sebastian Franco‐Tabares, Sarah A. Kuehne, Rachel Sammons

**Affiliations:** ^1^ Department of Prosthetic Dentistry/Dental Materials Science Institute of Odontology, Sahlgrenska Academy, University of Gothenburg Gothenburg Sweden; ^2^ School of Dentistry, College of Medical and Dental Sciences University of Birmingham Birmingham UK; ^3^ Institute of Microbiology and Infection University of Birmingham Birmingham UK

**Keywords:** biocompatible materials, biofilms, dental materials, polyetheretherketone

## Abstract

**Objective:**

Polyetheretherketone (PEEK) is a polymer used in devices in orthopedic and dental rehabilitation. The aim of this in vitro study was to compare biofilm formation by a range of important oral bacterial species on PEEK, blasted PEEK, commercially pure titanium (cp‐Ti), and titanium‐6 aluminium‐4 vanadium (Ti6Al4V).

**Material and methods:**

Coin‐shaped samples were manufactured, and the surfaces were characterized using optical interferometry, scanning electron microscopy, energy‐dispersive X‐ray spectroscopy, and contact angle measurements. Bacterial species of *Streptococcus sanguinis, Streptococcus oralis, Enterococcus faecalis*, and *Streptococcus gordonii* were cultured on the four material surfaces for varying amounts of time. Biofilms were quantified following staining with crystal violet.

**Results:**

Roughness and contact angle results showed blasted PEEK > PEEK > cp‐Ti = Ti6Al4V. There was increased biofilm formation on blasted PEEK by *S. sanguinis, S. oralis*, and *S. gordonii*, whereas the bacterial adhesion was similar on PEEK, cp‐Ti, and Ti6Al4V. The bacterial growth of *E. faecalis* was significantly higher on cp‐Ti compared with the other three groups.

**Conclusion:**

The results, taking into consideration the biofilm formation, suggest that PEEK should perform as well as cp‐Ti or TiAl6V4 when used as a dental restorative material.

## 1 INTRODUCTION

Polyetheretherketone, PEEK, is a biomaterial that has been on the market since the 1980s, and it is used as an alternative to metal‐based materials in orthopedics (e.g., spinal devices; Lied, Roenning, Sundseth, & Helseth, 2010), in maxillofacial surgery (e.g., bone reconstruction; Alonso‐Rodriguez et al., 2015), and more recently in dental rehabilitation (Najeeb, Zafar, Khurshid, & Siddiqui, 2016). It is a material with favorable biomechanical properties, and it can withstand chemical and biological degradation (Kurtz & Devine, 2007). In the field of oral prosthodontics, the interest in PEEK as a material in reconstructive applications has increased in the last years, though there have been few reported clinical studies. Titanium and zirconia are commonly used as framework materials in supraconstructions and abutments in implant dentistry. However, in recent years, other materials such as PEEK have also been used (Santing, Meijer, Raghoebar, & Özcan, 2012; Stawarczyk et al., 2013). Even though PEEK has many advantages, as with all biomaterials, undesired tissue reactions (e.g., allergic reactions) and infections may occur (Moriarty, Poulsson, Rochford, & Richards, 2011).

Biomaterial‐associated infections can cause serious complications, even though surgical‐site infections in patients who have had orthopedic implant surgery are rather rare. The incidence of prosthetic joint infections is approximately 0.4%, within 10 years of primary surgery and 2.3% after revision surgery, according to the National Joint Registry for England, Wales, Northern Ireland and the Isle of Man (Lenguerrand et al., 2017). In a review paper by Mombelli, Müller, and Cionca (2012), oral peri‐implant infections were reported to affect 10% of the implants and 20% of the patients, 5–10 years after implant placement. However, there were variations in prevalence of peri‐implantitis in the different studies, depending on factors such as different study designs, dissimilarities in the composition of study populations, and inconsistent disease definitions.

Biomaterials in the oral cavity are generally not sealed and protected in the tissue but instead exposed to the saliva with varying pH and to a wide variety of bacteria. More than 700 bacterial species have been detected in the oral environment (Aas, Paster, Stokes, Olsen, & Dewhirst, 2005), and biofilm formation occurs on all exposed surfaces including materials for restorative applications (Moons, Michiels, & Aertsen, 2009). The process of biofilm formation can be divided into three stages: attachment, colonization, and biofilm development (Hojo, Nagaoka, Ohshima, & Maeda, 2009). To survive in the oral cavity, bacteria must adhere to pellicle‐coated surfaces, desquamating surfaces or other bacteria that are already surface bound (Kolenbrander, Palmer, Periasamy, & Jakubovics, 2010). During attachment, initial colonizers utilize the pellicle generated by the saliva‐conditioning film and express surface receptors which facilitate their adherence to it (Periasamy & Kolenbrander, 2010). Primary colonizers include various *streptococci*, such as *Streptococcus sanguinis*, *Streptococcus gordonii*, and *Streptococcus oralis*, which can adhere directly to the surface and bind to other species in the initial biofilm (Kreth, Merritt, & Qi, 2009). Many of these can utilize sucrose and other carbohydrates from the diet to form polysaccharides, which contribute to the extracellular matrix, facilitating adhesion and colonization (Dahlen, 2009). Biofilms are found in healthy individuals and are usually harmless, consisting of predominantly commensal bacteria. However, if they are allowed to accumulate, the composition may change, allowing pathogens to become more prevalent (Øilo & Bakken, 2015). Depending on the location, caries, gingivitis, and subsequently periodontitis or peri‐implantitis may occur. Some pathogens, for example, Enterococcus faecalis, have been shown to be highly resistant to a range of antibiotics (Kouidhi, Zmantar, Mahdouani, Hentati, & Bakhrouf, 2011). Therefore, it is important to use materials that do not enhance biofilm formation.

The factors that determine the amount of bacterial growth on different restorative materials are not well understood. Some studies have shown that biofilm formation on metals differs from that on ceramics and polymers (Busscher, Rinastiti, Siswomihardjo, & Van der Mei, 2010). In addition to chemical composition and surface free energy, the presence and dimensions of surface features such as pores and defects, which can create favorable conditions for bacterial growth, may also influence bacterial adhesion (Øilo & Bakken, 2015). Hahnel, Wieser, Lang, and Rosentritt (2015) compared multispecies biofilms on different abutment materials in vitro and showed that biofilm formation on PEEK was equal or lower compared with zirconia and titanium. However, the PEEK material surface used in Hahnel's study was significantly smoother than both zirconia and titanium and this could have influenced the result, because studies have shown that an increase in surface roughness significantly favors bacterial attachment and biofilm formation and facilitates its growth (Bollen, Lambrechts, & Quirynen, 1997; Teughels, Van Assche, Sliepen, & Quirynen, 2006). Most studies on bacterial growth on PEEK have mainly focussed on pathogens associated with orthopedic infections such as Staphylococcus aureus, Staphylococcus epidermidis, Pseudomonas aeruginosa, and Escherichia coli (Barton, Sagers, & Pitt, 1996; Rochford et al., 2014). Increased knowledge of bacterial adhesion and biofilm formation on novel materials can help us improve our understanding regarding their applications and the potential risk of developing diseases. However, according to Hahnel et al. (2015), there is almost no available literature on the adhesion and proliferation of clinically relevant oral bacteria on PEEK. The objective of this in vitro study was to compare biofilm formation by different oral bacterial species on PEEK with different surface textures, that is, “as prepared” and blasted, in comparison with commercially pure titanium (cp‐Ti) and the most commonly used titanium alloy, titanium‐6 aluminum‐4 vanadium (Ti6Al4V). The hypothesis was that bacterial adhesion and biofilm formation would be affected by material composition and by the surface roughness.

## 2 MATERIALS AND METHODS

Four groups of materials were used: PEEK, blasted PEEK, cp‐Ti, and Ti6Al4V.

### 2.1 PEEK

The PEEK samples were machined from Ketron® Life Science Grade (LSG) natural PEEK (Quadrant EPP NV, Tielt, Belgium). One group of PEEK samples was left “as prepared,” and the other group was surface‐treated by abrasive grit blasting using 110‐μm aluminum oxide (Al2O3) particles at an air pressure of 2 bar, applying an airborne particle abrasion unit (Basic Quattro; Renfert GmbH, Hilzingen, Germany) for 10 s per side at a distance of 20 mm.

### 2.2 Commercially pure titanium

The samples were machined from Titanium Grade 4 according to ISO 5832‐2/ASTM F67 (Zapp Medical Alloys GmbH, Schwerte, Germany).

### 2.3 Titanium‐6 aluminum‐4 vanadium

The samples were machined from Ti6Al4V extra low interstitials according to material specification ISO 5832‐3/ASTM F136 (Xi'an Aerospace New Material Co., Ltd., Xi'an Xian, Shanxi, China).

All samples were machined from a rod with a diameter of 10 mm into 2‐mm‐thick coin‐shaped samples. The cutting speed was 94 m/min and the feed rate was 0.01–0.02 mm/rev. After machining, the coins were radially turned on both sides with a tool composed of solid carbide. Following this, the samples were ultrasonically cleaned with 1% Extran® AP15 (Merck, Darmstadt, Germany) in 60°C tap water for 15 min. Thereafter, the samples were rinsed in distilled water and immersed in 70% ethanol for 15 min. After drying, the samples were packed in sterilization pouches, which were sealed and sterilized in an autoclave (Getinge AB, Getinge, Sweden) at 134°C and 3 bar in a 60‐min program.

### 2.4 Topography

The topographical information was acquired using a white‐light interferometer (SmartWLI extended, Gbs, Germany). Three coins from each group were vertically scanned at three different sites using a 50× Mirau objective with a height resolution of 0.1 nm. For each scan, an antivibration device was activated (Nanoseries, Accurion, Germany), the upper and lower bounds of each scan were 250 μm approximately. The size of the measured area was 356 × 223 μm. The data was initially acquired using the SmartVIS3D software version 2.1 (Gbs, Germany) and processed using the MountainMaps software version 7.4 (Digital Surf, France). The processing of the data was performed in two steps. An initial removal of isolated outliers was performed and afterwards, a high‐pass Gaussian filter of size 50 × 50 μm was used to separate roughness from form and waviness (Wennerberg & Albrektsson, 2000). The following topographical parameters were measured: Sa (μm), which is the average roughness; average height deviation from a mean plane within the measuring area. Sds (1/μm^2^), which is the summit density; the number of summits per unit area. Sdr (%), which is the developed interfacial area ratio; additional surface area contributed by the roughness, as compared with a totally flat plane.

### 2.5 Scanning electron microscopy

Samples were mounted on aluminum stubs using carbon tabs. Copper tape was used to enhance conductivity of the PEEK samples and all samples were gold sputter coated at a deposition voltage of 25 kV for 2 min (Emitech K550X Sputter Coater, East Grinstead, UK). Images were acquired using a Zeiss Evo MA10 Scanning Electron Microscope (Cambridge, UK). Three specimens of each sample were imaged at magnifications ranging from 500× to 6,000×, at a working distance of 10 mm, at five or more separate locations across the entire sample.

### 2.6 Energy‐dispersive X‐ray spectroscopy

Energy‐dispersive X‐ray spectroscopy (EDX) analyses were performed to confirm the elemental composition of the samples and to check for contamination after blasting. The specimens were mounted on aluminum stubs (12.5 mm ø, AGG301, Agar Scientific, UK) and fixed using carbon adhesive discs (12 mm ø, AGG3347N, Agar Scientific, UK). A narrow line of silver paint (G3691, Agar Scientific, UK) was used to enhance the conductivity of the specimens. The specimens were gold‐coated (approximately 5.0 nm) using the Q150T ES coater (Quorum Technologies, UK). The EDX spectra were generated using a LEO Ultra 55 scanning electron microscope at 10 kV (Carl Zeiss, Germany) equipped with an EDX detector (Inca, Oxford, UK).

### 2.7 Contact angle

Droplets (5 μl in volume) of distilled water were applied onto each surface in three different areas and the contact angle imaged using a JVC‐3CCD video camera (JVC, Yokohama, Japan). Average water contact angles (θ) were calculated using Optimas 6.5 (Glenview, Illinois, USA) image analysis software.

### 2.8 Microbiological procedure

To compare bacterial growth and biofilm formation on the four materials, four species of bacteria, which are commonly found in oral biofilms were selected: *S*. *sanguinis* (ATCC 10556), *S*. *oralis* (ATCC 35037), E. faecalis (ATCC 19433), and *S*. *gordonii* (ATCC 10558). All bacteria were cultured on tryptone soya agar (Oxoid, Basingstoke, UK) and incubated in an atmosphere of 5% CO_2_ at 37°C for 24–48 hr to obtain single colonies. A single colony was suspended in 10 ml of brain heart infusion (BHI) broth (Oxoid, Basingstoke, UK) containing 1% sucrose (Fluka Analytical) and incubated overnight at 37°C/100 rpm in a shaking incubator (N‐BIOTEK NB‐205, Progen Scientific, London, UK). *S*. *gordonii* was similarly cultured in BHI in the presence of 1% sucrose and in its absence, for comparison of conditions which would be less conducive to polysaccharide synthesis. Overnight cultures were diluted with BHI to obtain a suspension containing approximately 10^3^ colony‐forming units (cfu)/ml and 1 ml of this was transferred to sterile coin samples in a 24 well plate (Thermo Fisher Scientific, Loughborough, UK). The plate was then incubated at 37°C/40 rpm, changing the media every 24 hr, for up to 120 hr. Each experiment consisted of four coins of each material for each time point. Three of the four coins were used for biofilm quantification and the remaining one was processed for scanning electron microscopy (SEM), as described below.

### 2.9 Biofilm assay

Biofilm formation was quantified by a modification of the method of Christensen et al. (1985). Briefly, the samples were transferred to a new 24‐well plate and rinsed once with phosphate‐buffered saline (PBS). Fixation was carried out by immersing the samples in 1 ml of 10% formalin for 5 min followed by rinsing with PBS. Following this, 300 μl of 0.1% crystal violet stain (Prolab‐diagnostics, Bromborough, UK) was then added for 5 min, removed, and samples rinsed three times with PBS to remove excess stain. They were then dried for 2 hr at 37°C. The crystal violet was solubilized by immersion in 200 μl methanol on a shaking table for 2 hr. The samples were removed, and the absorbance read at 590 nm using a spectrophotometer (Jenway 7315, Staffordshire, UK). The absorbance of the eluted stain is proportional to the concentration of bacteria present on the sample surface.

### 2.10 SEM of biofilms

Following biofilm formation, the culture medium was removed, and samples were rinsed in PBS. They were then fixed by addition of 2.5% glutaraldehyde (electron microscopy (EM) grade, Agar Scientific, UK) in 0.1 M sodium cacodylate buffer (Sigma UK) pH 7.2 for 10 min at room temperature, followed by dehydration in an ethanol series of increasing concentration from 20%–100%. The 100% ethanol was replaced by hexamethyldisilizane (Sigma, UK), which was removed by evaporation. The samples with biofilms were acquired with the same SEM methodology as described earlier.

### 2.11 Statistical analysis

Statistical comparisons of the interferometry, the contact angle measurements, and the biofilms were carried out using one‐way analysis of variance (ANOVA) followed by Tukey's post hoc test. Two‐way ANOVA followed by Tukey's post hoc test was used to compare the effect of the two independent variables (i.e., material and time) on biofilm formation. Statistical significance was evaluated using Minitab 17statistical software (State College, Pennsylvania, USA) or SPSS ver. 21.0 software (SPSS IBM, Chicago, IL, USA). A *p* value <.05 was considered statistically significant.

## 3 RESULTS

### 3.1 Topography

The results from the surface topography measurements are presented in Table 1. The blasted PEEK surface was significantly rougher and showed higher mean values of all parameters (Sa, Sds, Sdr) compared with the other three surfaces. Both cp‐Ti and Ti6Al4V surfaces were significantly smoother than the PEEK surfaces and showed lower mean Sa and Sds values.

**Table 1:** Results of the surface topography analysis

Material	Sa μm	Sds 1/μm^2^	Sdr %
PEEK	0.57 (0.08)^a^	0.37 (0.02)^a^	14.25 (7.01)^a^
Blasted PEEK	1.85 (0.19)^b^	0.53 (0.02)^b^	167.11 (55.03)^b^
cp‐Ti	0.23 (0.01)^c^	0.28 (0.01)^c^	2.23 (0.29)^a^
Ti6Al4V	0.28 (0.01)^c^	0.28 (0.02)^c^	1.56 (0.41)^a^

Abbreviations: cp‐Ti, commercially pure titanium; PEEK, polyetheretherketone; Ti6Al4V, titanium‐6 aluminum‐4 vanadium.

Mean values for surface topography parameters Sa, Sds, and Sdr. Standard deviations are given in parentheses. Within a column, means that do not share a superscript letter are significantly different. Tested by one‐way analysis of variance followed by Tukey's post hoc test, significance level set at *p* < .05.

### 3.2 Scanning electron microscopy

**Figure 1:**
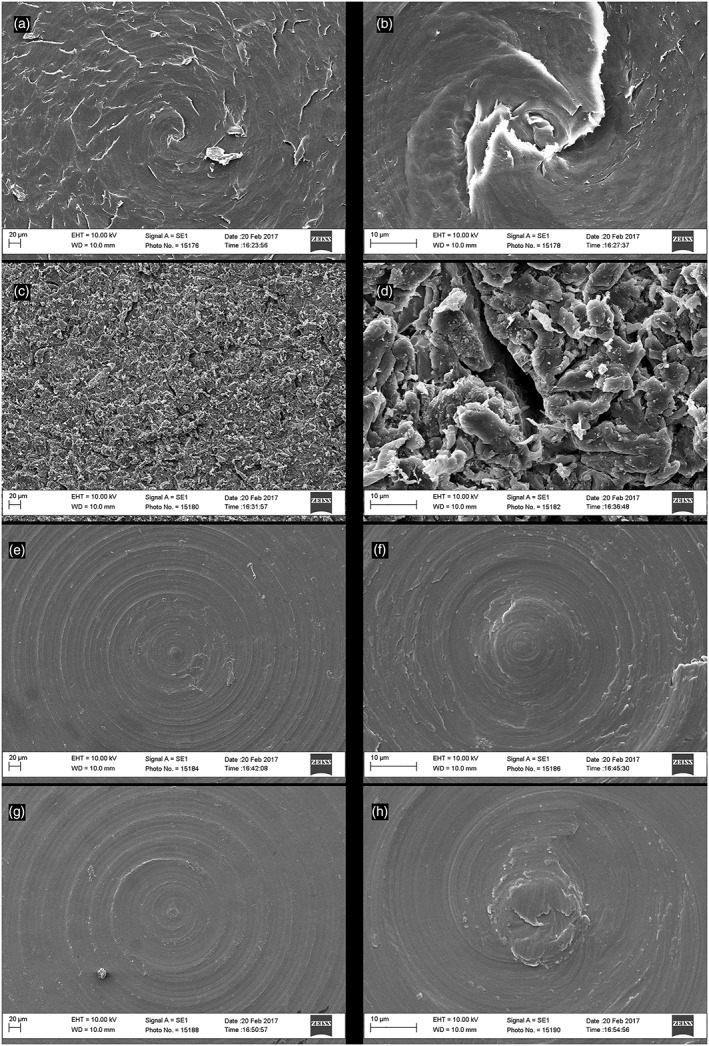
Scanning electron microscopy micrographs of: (a and b) polyetheretherketone (PEEK), (c and d) Blasted PEEK, (e and f) commercially pure titanium, and (g and h) titanium‐6 aluminum‐4 vanadium. Images on left, bar = 20 μm and zoomed images on right, bar = 10 μm

SEM images of the coin surfaces are shown in Figure 1. Micrographs obtained from the center of each sample reveal circular milling lines except for the blasted PEEK. Both titanium surfaces appear relatively smooth except for these milling lines (Figure 1e–h), whereas the PEEK surface shows some irregular roughness (Figure 1a–b). The blasted PEEK surface differs considerably with the entire surface being roughened with micron‐scale peaks and pits (Figure 1c,d).

### 3.3 Energy‐dispersive X‐ray spectroscopy

The results from the EDX analysis confirmed the expected purity of cp‐Ti and the composition of Ti6Al4V (Table 2). The presence of a small amount of Al in the blasted PEEK sample may be due to remnants of the Al_2_O_3_ blasting media lodged in the surface.

**Table 2:** Results of the EDX analysis

	Atomic %				
	C	O	Al	V	Ti
PEEK	82.00 (2.20)	16.61 (2.50)	nd	nd	nd
Blasted PEEK	81.98 (3.49)	15.73 (3.60)	0.90 (0.96)	nd	nd
cp‐Ti	nd	nd	nd	nd	96.97 (0.07)
Ti6Al4V	nd	nd	9.86 (0.64)	2.33 (0.74)	85.00 (0.47)

Abbreviations: cp‐Ti, commercially pure titanium; EDX, energy‐dispersive X‐ray spectroscopy; PEEK, polyetheretherketone; nd, not detected; Ti6Al4V, titanium‐6 aluminum‐4 vanadium.

Mean values of the atomic composition of the four materials determined with EDX. Standard deviations are given within parentheses.

### 3.4 Contact angle

The results of the contact angle measurements are presented in Table 3. The blasted PEEK showed the highest mean contact angle. Both PEEK samples were significantly more hydrophobic than the titanium samples (<0.01), but there was no statistical difference between cp‐Ti and Ti6Al4V (0.06). Blasted PEEK was significantly more hydrophobic than the nonblasted PEEK (<0.001).

**Table 3:** Contact angle measurements for the different materials

Material	Water contact angle, θ (°)
PEEK	70.33 (1.57)^a^
Blasted PEEK	108.36 (2.48)^b^
cp‐Ti	62.43 (1.45)^c^
Ti6Al4V	58.82 (1.92)^c^

Abbreviations: cp‐Ti, commercially pure titanium; PEEK, polyetheretherketone; Ti6Al4V, titanium‐6 aluminum‐4 vanadium.

Mean contact angles with standard deviation for the drop between the liquid and solid (°). *N* = 3. Means that do not share a superscript letter are significantly different. Tested by one‐way analysis of variance followed by Tukey's post hoc test, significance level set at *p* < .05.

### 3.5 Biofilm formation

**Figure 2:**
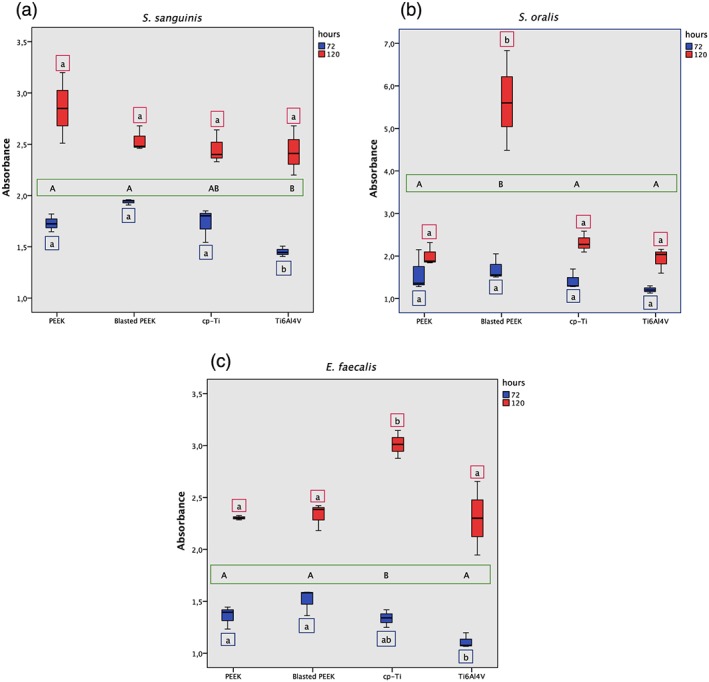
Biofilm formation of (a) Streptococcus sanguinis, (b) Streptococcus oralis, (c) Enterococcus faecalis. The box plot shows crystal violet 590 nm absorbance at 72 hr (blue) and 120 hr (red). Medians at 72 hr and 120 hr that do not share a lower case letter are significantly different, tested with one‐way analysis of variance (ANOVA) p < .05 followed by Tukey's post hoc test. Significant differences between materials tested with two‐way ANOVA followed by Tukey's post hoc test (p < .05). Medians that do not share an uppercase letter are significantly different

*S*. *sanguinis* showed the highest biofilm formation on blasted PEEK at 72 hr, but there were no significant differences compared with PEEK and cp‐Ti (Figure 2a). There was significantly less biofilm on Ti6Al4V when compared with the other three materials after 72 hr. As expected, there was an increase in biofilm after 120 hr on all surfaces, but the results were more variable and there were no significant differences between the groups after this time. A two‐way ANOVA was performed to determine the differences between the sampled groups with the interaction of time and material. The analysis showed that PEEK and blasted PEEK had significantly higher mean absorbances compared with Ti64AlV, whereas cp‐Ti did not differ from any of the other groups.

**Figure 3:**
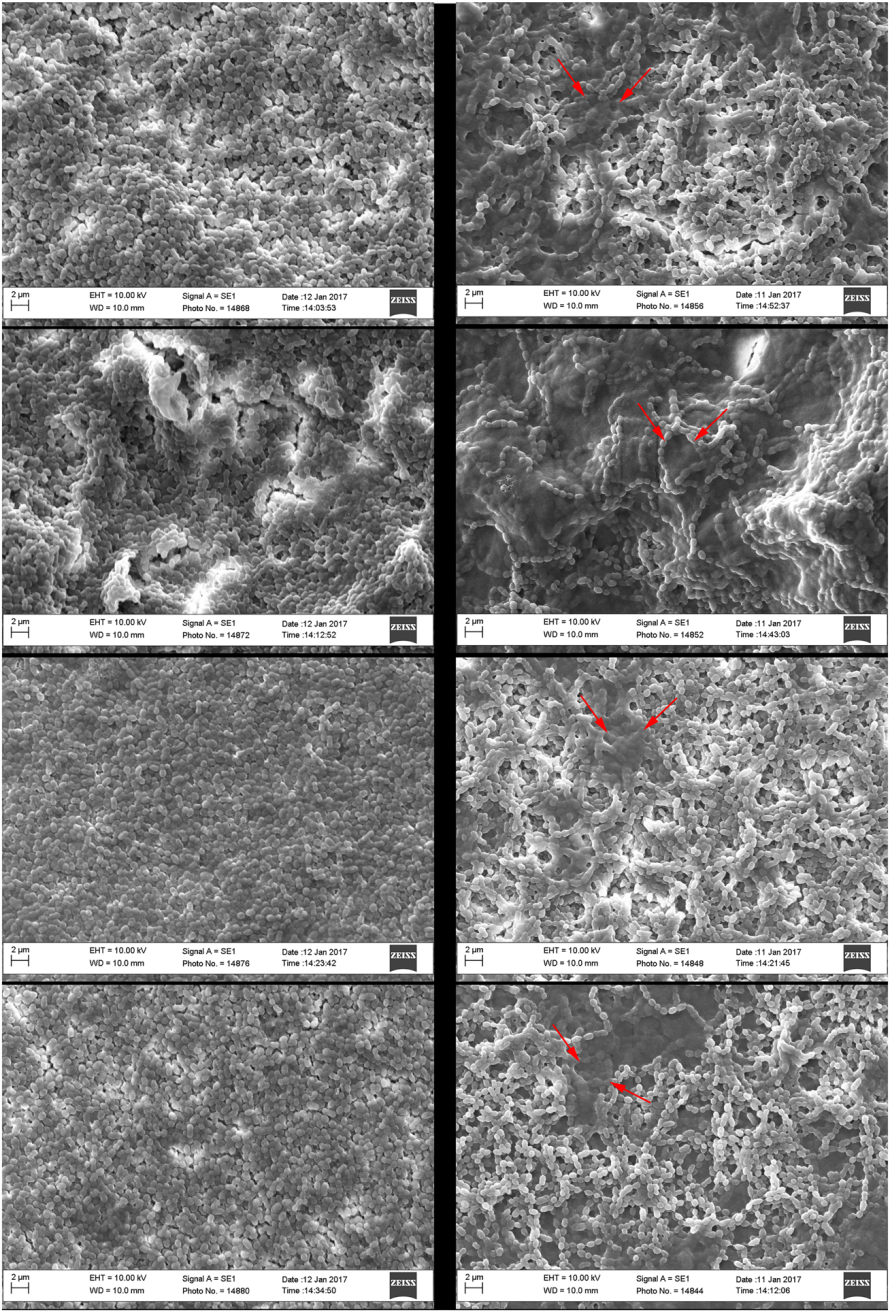
Scanning electron microscopy micrographs of Streptococcus sanguinis biofilms at 72 hr (left) and 120 hr (right), top to bottom: polyetheretherketone (PEEK), blasted PEEK, commercially pure titanium, and titanium‐6 aluminum‐4 vanadium. Biofilms are clearly present on all surfaces. Some of the bacteria in the images especially at 120 hr are obscured by a matrix‐like substance (dark patches) around the streptococci. Bar = 2 μm in all images

*S*. *sanguinis* completely covered the surfaces at both 72 and 120 hr (Figure 3). After 120 hr, some of the cells were obscured by an amorphous extracellular matrix‐like substance. Similar appearances were seen with the other bacteria at these times (not shown).

*S*. *oralis* showed no significant difference in biofilm formation on the different materials and surfaces after 72 hr (Figure 2b). However, after 120 hr, the blasted PEEK showed significantly higher absorbance (<0.05).

With E. faecalis, there was significantly more biofilm on PEEK and blasted PEEK compared with Ti6Al4V after 72 hr (Figure 2c). After 120 hr, the biofilm formation was significantly higher on cp‐Ti compared with the other three materials, as confirmed by two‐way ANOVA (<0.05).

**Figure 4:**
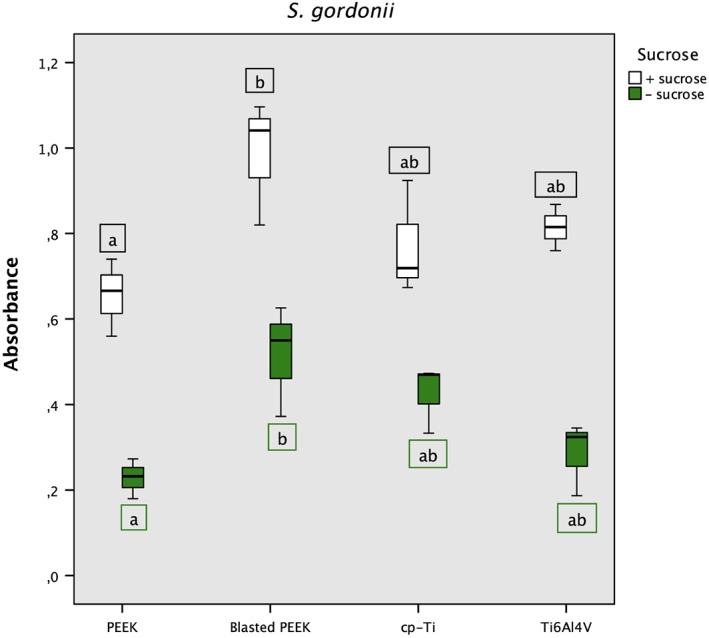
Biofilm formation of Streptococcus gordonii at 48 hr. The box plot shows crystal violet 590 nm absorbance in the presence (white) and absence of 1% sucrose (green). Medians that do not share a lower case letter are significantly different, tested with one‐way analysis of variance p < .05 followed by Tukey's post hoc test

**Figure 5:**
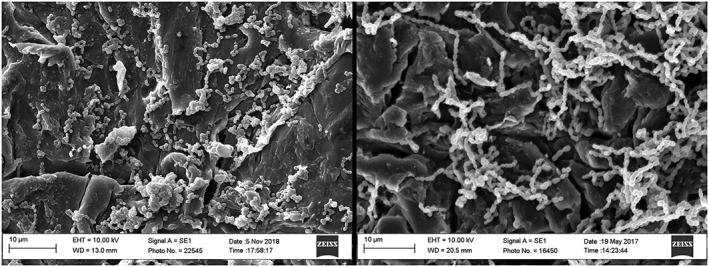
Streptococcus oralis (left) and Streptococcus gordonii (right) biofilm on blasted polyetheretherketone at 48 hr. Some bacterial chains can be seen lying within cracks and crevasses. Bar = 10 μm

*S*. *gordonii* biofilm formation was greater on all four materials when grown in the presence of sucrose, compared with its absence (Figure 4). Extracellular amorphous material, presumed to be polysaccharide, appeared in SEM images of all strains after longer culture periods and, as expected, was less abundant in *S*. *gordonii* samples grown in the absence of sucrose. Under both conditions, there was significantly more biofilm on blasted PEEK than on PEEK. SEM images of *S*. *oralis* and *S*. *gordonii* on the rougher blasted PEEK surface (Figure 5) show chains of bacteria lying within the cracks and crevices. There was no significance difference in the amount of biofilm on cp‐Ti and Ti6Al4V.

## 4 DISCUSSION

The results of this in vitro study showed that all the bacteria grew well on all materials and are similar to those of Barton et al. (1996), who showed that bacteria adhered equally well to a range of orthopedic polymers. However, there were significant differences in the bacterial growth between the different material groups. Our hypothesis was that the biofilm formation would be affected by the various materials' composition and roughness. The growth of *S*. *sanguinis* was lower on the Ti6Al4V compared with the other groups at 72 hr, but after 120 hr, there were no differences between the groups, possibly because by this point growth of the population has slowed down due to competition for space and nutrients.

We could confirm that the surface roughness had an impact on the bacterial adhesion to these materials. When comparing the effects of both material and time, the biofilm formation for *S*. *sanguinis* was significantly higher on PEEK and blasted PEEK compared with Ti6Al4V. *S*. *oralis* also grew to a higher extent on the blasted PEEK compared with all the other groups. The same tendency could be seen with *S*. *gordonii*, where the blasted PEEK had significantly higher biofilm formation compared with PEEK, both in the presence and absence of sucrose. It is known that increased surface roughness increases the amount of bacteria in the biofilm compared with a smoother surface (Teughels et al., 2006). One reason for this is that the bacteria can attach easier and become sheltered in the small micrometer scale cracks in the rougher surface (Bollen et al., 1997), as can be seen in the SEM images in Figure 5.

There is also a greater area for the attached cells to grow on as indicated by the parameter Sdr, which was significantly higher on the blasted PEEK. Sdr describes the additional surface area contributed by roughness, as compared with a totally flat plane. For implanted materials that are intended to interface with bone tissue, it is desirable to create a rough surface to encourage tissue ingress into the surface. However, for nonimplanted components, such as dental abutments, from an infection prevention point of view, it is therefore of importance to keep biomaterials in the oral cavity as smooth as possible, when aiming to decrease the amount of biofilm formation on the surface and for ease of cleaning. The milling lines present on the samples will slightly increase the surface area available for colonization and could help to anchor the confluent biofilm. However, their presence is unlikely to significantly affect initial bacterial adhesion because they are so much larger than the bacterial cells.

The wettability of a biomaterial has also been proposed to influence the biofilm formation (Wassmann, Kreis, Behr, & Buergers, 2017). Materials that have higher surface free energy will create a more wettable surface and are more likely to adhere bacteria (Teughels et al., 2006), although this depends on the hydrophobicity of the bacteria (Song, Koo, & Ren, 2015). However, in the present study, the blasted PEEK had the highest contact angle (least wettable) followed by PEEK. A possible explanation for this is that air is trapped in the spaces between the protruding surface features, repelling the water droplet, as has been seen with many similarly rough surfaces (Bico, Thiele, & Quéré, 2002). Both *S*. *sanguinis* and *S*. *oralis* are reported to be hydrophobic, and they have previously shown to preferentially adhere to hydrophobic surfaces (Song et al., 2015). This could be a contributory factor in explaining their apparent affinity for PEEK blasted surfaces, in addition to increased surface area.

The chemical composition on the surface of the material may also play a role in biofilm formation (Auschill et al., 2002). The attachment of bacteria to different surfaces involves complex mechanisms with different chemophysical forces that will attract or repel bacteria (Teughels et al., 2006). The EDX analyses showed that blasted PEEK had traces of aluminum that was most likely derived from the airblasting of the PEEK surface with Al_2_O_3,_ but this does not have significant antimicrobial activity (Jastrzębska, Karwowska, Olszyna, & Kunicki, 2013) and probably had no effect on biofilm formation. As yet, there are only few studies on the effect of the surface chemistry on PEEK related to bacterial growth. Because surface composition and roughness influence wettability, it is difficult to determine which is the most influential. However, even though the degree of wettability and the surface roughness interact with each other, evidence suggests that surface roughness is the most important one of the two mentioned parameters (Quirynen & Bollen, 1995). In order to compare the effects of surface composition alone, it would have been possible to equalize the surface roughness on Ti and PEEK surfaces, and we acknowledge this as a weakness in this study, which should be addressed in further work. Nevertheless, although material composition may play a role, the larger surface area created by the porous surface is likely to be the more influential parameter because it affords a greater surface area and bacteria become entangled and trapped in the surface irregularities, as shown in Figure 5. After 72–120 hr, surface topography is minimized by a confluent layer of bacteria filling up the cracks and crevasses on all surfaces (Figure 3).

Our findings suggest that machined PEEK is no more susceptible to bacterial colonization than cp‐Ti or TiAl6V4 and from this point of view is a suitable alternative to metals in the prosthetic dentistry. Similar results were shown when comparing biofilm formation of S. aureus and S. epidermidis on PEEK and Ti (Rochford et al., 2014). In the present study, we saw similar amounts of bacterial growth on both cp‐Ti and Ti6Al4V for the initial colonizers *S*. *sanguinis*, *S*. *oralis*, and *S*. *gordonii*. This was expected because these two materials have similar roughness and wettability (Mabboux, Ponsonnet, Morrier, Jaffrezic, & Barsotti, 2004) and is consistent with results on *S*. *sanguinis* reported by Wang et al. (2018). However, there was significantly increased growth of E. faecalis on the cp‐Ti compared with the other three surfaces. Further work is needed to confirm this. This may be related to the bacterial surface properties and sensitivity to Al or V components in the alloy. Barão et al. (2014), observed higher attachment of *Porphyromonas gingivalis* to cp‐Ti than Ti6Al4V. They suggest that the reduced attachment of *P*. *gingivalis* on Ti6Al4V could be explained by the antimicrobial effect of vanadium, as reported by Tousley, Wren, Towler, and Mellott (2012). However, Wang et al. (2018) observed higher adhesion of E. coli, S. epidermidis, and *S*. *sanguinis* to pure V compared with pure Al. To date, the limited number of in vitro studies comparing bacterial growth on cp‐Ti and Ti6Al4V show inconsistent results, depending on the bacterial species and study design. Inconsistencies in results may also be due to heterogeneity in the cell wall properties of subpopulations of cells within a single species bacterial population, as reported for E. faecalis (van Merode, van der Mei, Busscher, & Krom, 2006). In a review article, Shah, Trobos, Thomsen, and Palmquist (2016) concluded that there is also a lack of in vivo studies regarding the bacterial growth and differences between cp‐Ti and Ti6Al4V.

Biomaterials introduced into the oral environment are immediately covered with a thin layer of pellicle, which consists of several proteins, enzymes, and other molecules from the saliva to which bacteria can attach to form a biofilm (Teughels et al., 2006). On the one hand, the saliva facilitates bacterial adhesion, but on the other hand, it also contains antibacterial proteins that inhibit bacterial growth and adhesion (Hannig & Hannig, 2009). Furthermore, the pellicle‐conditioning film may equalize differences in physicochemical surface properties to a certain extent (Hannig & Hannig, 2009). In the present study, the bacteria were not cultured in the presence of saliva, and thus its effect is unaccounted for. Another important aspect to consider is that biofilms within dental plaque do not consist of single species but rather build up in communities where several different species interact in a complex manner and respond to environmental changes as a single unit (Marsh, 2004). Even though single species were examined separately in the present study, the early colonizers *S*. *sanguinis, S*. *oralis*, and *S*. *gordonii* are of importance because they enable attachment of subsequent colonizers and therefore influence the composition of the maturing biofilm. Further studies will involve more complex, multispecies biofilms in the presence of saliva.

## 5 CONCLUSIONS

Within the limitations of this in vitro study, it can be concluded that bacterial adhesion was similar on PEEK, cp‐Ti, and Ti6Al4V. However, blasted PEEK with a rougher surface topography showed increased biofilm formation by *S*. *sanguinis*, *S*. *oralis*, and *S*. *gordonii*.
